# High Level of Soluble CD146 In Cerebrospinal Fluid Might be a Biomarker of Severity of Anti-N-Methyl-D-Aspartate Receptor Encephalitis

**DOI:** 10.3389/fimmu.2021.680424

**Published:** 2021-06-16

**Authors:** Qing Li, Jinglong Chen, Mengzhuo Yin, Jun Zhao, Fuchang Lu, Zhanhang Wang, Xiaoqi Yu, Shuangyan Wang, Dong Zheng, Honghao Wang

**Affiliations:** ^1^ Department of Geriatric Medicine, Guangzhou First People’s Hospital, South China University of Technology, Guangzhou, China; ^2^ Department of Neurology, Guangdong 999 Brain Hospital, Guangzhou, China; ^3^ Department of Neurology, The Affiliated Brain Hospital of Guangzhou Medical University, Guangzhou, China; ^4^ Department of Neurology, Nanfang Hospital, Southern Medical University, Guangzhou, China

**Keywords:** anti-NMDAR encephalitis, soluble CD146, cerebrospinal fluid, blood–brain barrier, neurological damages

## Abstract

**Background:**

Disruption of the blood–brain barrier (BBB) is an important pathophysiological process of anti-N-methyl-D-aspartate receptor (anti-NMDAR) encephalitis. A recent multi-center study showed that soluble (s) CD146 is a potential biomarker for monitoring early BBB damage and central nervous system inflammation, but little is known about sCD146 in anti-NMDAR encephalitis.

**Method:**

Twenty-three anti-NMDAR encephalitis patients and seventeen controls with non-inflammatory neurological diseases were recruited. sCD146 and inflammatory cytokines in cerebrospinal fluid (CSF) and serum were detected by ELISA. Modified Rankin scale (mRS) scores were used to assess the neurological status of each patient. A follow-up review was completed three months after discharge.

**Results:**

sCD146 levels in the CSF of patients with the acute stage anti-NMDAR encephalitis were significantly increased compared with controls and accompanied by increases in TNF-α, IL-6 and IL-10. CSF sCD146 was positively correlated with neuroinflammatory factors in the CSF and with mRS score. Three months after effective treatment, CSF sCD146 in patients was significantly decreased but remained significantly different compared with the controls.

**Conclusion:**

Our data suggested that higher expression of CSF sCD146 correlated with more serious neurological damage. Therefore, levels of CSF sCD146 may represent a promising indicator for monitoring disease and optimizing clinical treatment decisions in the early stages of anti-NMDAR encephalitis.

## Introduction

Anti-N-methyl-D-aspartate receptor (anti-NMDAR) encephalitis has gradually become recognized as an autoimmune disease targeting neuronal synapses. Generally, neuropsychiatric symptoms, including cognitive dysfunction, seizure, abnormal movements, autonomic instability and hypoventilation, are the most classic clinical features to be presented (1–4). Although anti-NMDAR antibodies can be found in the serum and cerebrospinal fluid (CSF) of patients with anti-NMDAR encephalitis, disruption of the blood–brain barrier (BBB) in anti-NMDAR encephalitis remains unclear.

CD146 is an adhesion molecule which was discovered in 1987 in the plasma membrane of human melanoma cells (5, 6). In recent decades, CD146 has been shown to be a type of transmembrane glycoprotein primarily expressed at the intercellular junctions of endothelial cells in the blood vessels of the central nervous system (CNS), and plays a key role in controlling the permeability and integrity of vessels (5–7). The soluble form of CD146 (sCD146) comes from shedding of the extracellular portion of CD146 via matrix metalloproteinases (MMPs) (5–7). Studies have proven that MMPs are elevated in anti-NMDAR encephalitis (8, 9), and to date, higher sCD146 has been found in chronic obstructive pulmonary disease (10), active inflammatory bowel disease (11), systemic sclerosis (12, 13), chronic renal disease (14, 15) and CNS diseases including multiple sclerosis (MS) (16, 17), neuromyelitis optica (16), CNS infections, peripheral neuropathy and Alzheimer’s disease (4–6, 18, 19). BBB integrity and low permeability are necessary for maintaining normal function of the CNS, and BBB disruption may play an important role in anti-NMDAR encephalitis (16, 18, 20). Thus, based on a recent multi-center study in MS with large samples conducted by Wang et al. (16), the results showed that compared with other soluble adhesion molecules, sCD146 may become a potential biomarker as it has high sensitivity and specificity for evaluating early BBB damage and CNS inflammation. Currently, little is known about sCD146 in anti-NMDAR encephalitis. In this study, we measured the levels of sCD146 in the CSF and serum of patients with anti-NMDAR encephalitis and assessed its potential clinical value in the diagnosis and treatment of this disease.

## Materials and Methods

### Patients and Controls

In this study, twenty-three patients who were diagnosed with anti-NMDAR encephalitis from 2018 to 2020 according to the revised anti-NMDAR encephalitis diagnosis criteria of 2016 were recruited from the Department of Neurology, The Affiliated Brain Hospital of Guangzhou Medical University, Guangzhou, China. The diagnosis of anti-NMDAR encephalitis was confirmed based on clinical manifestations and positive identification of antibodies against the NRI subunit of NMDAR in the CSF by cell-based analysis. Seventeen patients with non-inflammatory neurological diseases were selected as controls, including seven cases of Parkinson’s disease and ten cases of normal pressure hydrocephalus. Both anti-NMDAR encephalitis patients and controls were negative for the detection of common viruses and other pathogens by PCR. The study was approved by the Ethics Committee of The Affiliated Brain Hospital of Guangzhou Medical University. All participants provided informed consent before proceeding with the subsequent study. CSF samples from patients and controls were collected by lumbar puncture within three days of admission for diagnosis; blood samples were collected at the same time. CSF and blood samples were processed within 30 min of collection and centrifuged at 4000 rpm for 5 min. Supernatants of the CSF and serum samples were separated as soon as possible, transferred, numbered and stored at −80°C until they were used in ELISAs. The integrity of the BBB was evaluated by calculating the CSF to serum albumin quotient (QAlb), which represented an approximation of BBB breakdown. Modified Rankin Scale (mRS) scores were used to assess the neurological status of each patient. In addition to tumor removal, all patients were treated with first-line immunotherapy comprising intravenous methylprednisolone (1000 mg for 3–5 days), intravenous immunoglobulin (IVIg) (1 g/kg for 5 days per cycle) or plasma exchange, alone or combined (21, 22). Three months after discharge, patients were followed up and CSF and serum samples were obtained, along with reassessment by mRS.

### Detection of sCD146 by ELISA

Commercial sandwich ELISA kits were used to detect the inflammatory cytokines tumor necrosis factor-α (TNF-α) (Cusabio, Wuhan, China), matrix metalloproteinase-2 (MMP-2), interleukin-6 (IL-6), IL-10 and sCD146 (Bender Med-Systems GmbH Campus, Vienna, Austria) in the CSF and serum. Assays were performed according to the manufacturer’s instructions.

### Statistical Analysis

Descriptive statistics were performed on demographic factors and clinical features of patients. The Kruskal-Wallis test was used to analyze the differences in TNF-α, MMP-2, IL-6, IL-10, and sCD146 levels among subgroups. Pearson’s test or Spearman’s test were used to evaluate the correlations between TNF-α, MMP-2, IL-6, IL-10, sCD146 and mRS scores. A value of P<0.05 was considered statistically significant, and all statistical analyses were conducted using SPSS version 17.0 (IBM Corp, Armonk, NY).

## Results

### Patient Characteristics

Detailed clinical features and levels of inflammatory cytokines in CSF or serum collected from patients with anti-NMDAR encephalitis (n=23) and controls with non-inflammatory neurological diseases (n=17) are presented in **Table 1**. Only two anti-NMDAR patients (8.7%) had a history of teratomas. Psychiatric symptoms (82.6%) was the most common clinical presentation. All patients had moderate to severe disability with mRS from 3–5. Ten patients (43.78%) had high QAlb values. The degree of BBB damage was calculated according to QAlb×10^3^, where values <6 indicate no BBB damage; 6–8, mild; 8.1–10, moderate; >10, severe damage. Mild dysfunction was detected in four patients, moderate in two and severe in four. IVIg (69.57%) and steroids (78.26%) were the major treatments.

**Table 1:** Clinic characteristics of anti-NMDAR encephalitis patients and controls.

	Anti-NMDAR Encephalitis (n = 23)	Control (n = 17)	p value
**Gender (male/female)**	8/15	8/9	0.000
**Age (years, mean ± SD)**	29.6±14.2	27.5±9.9	0.208
**Clinic symptoms (n, %)**			
Fever	1 (4.4)	0	“-
Psychiatric symptom	19 (82.6)	0	“-
Disorders of memory	2 (8.7)	0	“-
Seizure	12 (52.2)	0	“-
Abnormal movements	1 (4.4)	0	“-
Automatic instability	3 (13.1)	0	“-
Hypoventilation	0 (0)	0	“-
**CSF routine (mean ± SD)**			
WBC (×10E6/L)	7.8±25.7	2.0±2.2	0.000
GLU (mmol/l)	3.7±0.6	3.7±0.6	0.924
Cl (mmol/L)	116.5±6.5	125±3.4	0.000
**Treatment (n, %)**			
Plasma exchange	5 (21.7)	“-	“-
IVIg	16 (69.6)	“-	“-
Steroids	18 (78.3)	“-	“-
**Qalb (mean ± SD, damage %)**	6.4±3.8 (43.5)	4.445±1.167 (11.8)	0.001
**CSF anti-NMDAR antibody positive (n, %)**	23 (100)	0	“-
**Tumor comorbidity (n, %)**	2 (8.7)	0	“-
**Max mRS**			
3 (n, %)	8 (34.8)	“-	“-
4 (n, %)	10 (43.5)	“-	“-
5 (n, %)	5 (21.7)	“-	“-

### sCD146 Was Increased in the CSF of Anti-NMDAR Encephalitis in the Acute Stage and Decreased After Three Months of Treatment

The expression of sCD146 in CSF of patients was notably increased in anti-NMDAR encephalitis patients in the acute stage compared with controls (P<0.001) but no difference was shown in serum sCD146 between these two groups. The QAlb and CSF MMP2 of anti-NMDAR patients were also significantly higher than those in the control group, as were the levels of CSF TNF-α, IL-10, and IL-6 (P<0.001).

**Figure 1:**
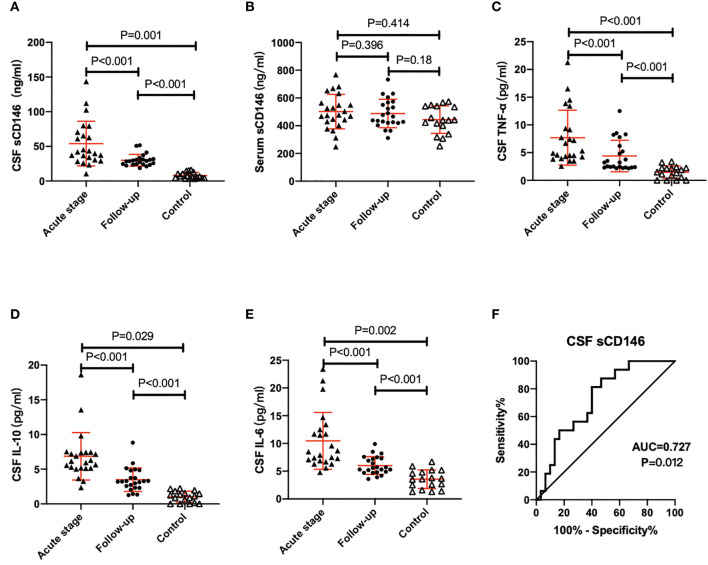
sCD146 levels in the CSF of patients with acute stage anti-NMDAR encephalitis were significantly increased compared with controls and 3-month follow-up accompanied by increases in TNF-α, IL-6 and IL-10 **(A–E)**. The ROC curve of CSF sCD146 to predict the severity of neurologic impairments in anti-NMARD encephalitis patients **(F)**.

All patients were followed up for three months after receiving different treatments. CSF sCD146 levels were significantly decreased from 54.0±32.2 to 29.9±8.5 (ng/ml) (P=0.001), and TNF-α, IL-10 and IL-6 in CSF were also decreased significantly (**Table 2**). Despite this, both sCD146 and inflammatory factors remained significantly elevated in anti-NMDAR patients compared with controls (**Figure 1**). However, neither serum nor CSF levels of MMP2 in the follow-up differed from levels detected during the acute stage. The mRS of all patients dropped below 3 at follow-up.

**Table 2:** Expression of inflammatory factors in the CSF and serum of anti-NMDAR encephalitis patients at the acute stage and 3-month follow up.

	Acute stage	Follow up	p value
**TNF-α (pg/ml)**	7.7±5.0	4.4±2.9	0.000
**IL-10 (pg/ml)**	6.9±3.4	3.5±1.7	0.000
**IL-6 (pg/ml)**	10.5±5.1	6.0±1.6	0.000
**Serum MMP2 (ng/ml)**	241.3±90.8	232.6±84.7	0.621
**CSF MMP2 (ng/ml)**	36.6±15.0	31.9±13.1	0.170
**Serum sCD146 (ng/ml)**	501.8±124.5	488.0±102.2	0.396
**CSF sCD146 (ng/ml)**	54.0±32.2	29.9±8.5	0.001
**mRS**	3.9±0.8	1.8±1.1	0.000

### CSF sCD146 Was Positively Correlated With Clinical Parameters of Anti-NMDAR Encephalitis Patients in the Acute Stage, Including Multiple Inflammatory Factors and mRS

**Figure 2:**
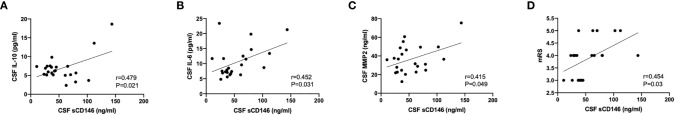
CSF sCD146 was positively correlated with neuroinflammatory factors in the CSF and with mRS score **(A–D)**.

As shown in **Figure 2**, significant positive correlations were found between sCD146 levels and IL-10 (r=0.479, p=0.021), IL-6 (r=0.452, p=0.031), and MMP2 (r=0.415, p=0.049) in the CSF of anti-NMDAR encephalitis patients in the acute stage. No significant correlations were shown between CSF sCD146 and inflammatory factors in patients at the 3-month follow-up or in the non-inflammatory disease control group.

There was a significant positive correlation between CSF sCD146 levels and mRS scores at the acute stage (r=0.417, p=0.004) (**Figure 2D**). To evaluate the ability of CSF sCD146 to indicate the severity of neurologic impairments in patients with anti-NMDAR encephalitis, we further performed receiver operating characteristic curve (ROC) analysis (**Figure 1F**). Here we defined the severity according to mRS scores (3, moderate disability; >3, severe disability). The area under curve was 0.727 (p=0.012; cut-off value, 33.75 ng/ml; Youden index, 0.418)

## Discussion

In our study, CSF and serum of twenty-two anti-NMDAR encephalitis patients and seventeen controls with non-inflammatory neurological diseases were examined. We found that sCD146 levels were markedly elevated in the CSF of anti-NMDAR encephalitis patients. Moreover, this biomarker was significantly associated with disease severity. To our knowledge, this study is the first to report changes in sCD146 levels in the CSF of patients suffering from this disease.

Disruption of the BBB is present in a variety of neuroinflammatory diseases. In a previous study of in vitro BBB and mouse models, Chen et al. (7) reported that CD146 was expressed universally in different regions of the brain and played a vital role in the formation and function of the BBB. In a recent study, Wang et al. (16) showed that CSF sCD146 directly promoted BBB hyperpermeability in active MS. sCD146 was found not only to be a sensitive marker for BBB damage but also a novel driver of neuroinflammatory dysfunction (5, 16, 23). Therefore, we speculated that there was significant BBB dysfunction in patients with acute anti-NMDAR encephalitis. Endothelial cells of the BBB are more easily damaged under this inflammatory state while the upregulation of sCD146 can impair the permeability of the BBB and thus aggravate disease severity.

QAlb is often used as a common indicator to evaluate destruction of the BBB. In this study, we found that the mean QAlb of anti-NMDAR encephalitis patients was abnormally high compared with controls, suggesting impairment of the BBB. Despite QAlb being elevated in 43.48% (10/23) of patients, no significant correlation was found between QAlb and CSF sCD146. Similar to our data, Wu, et al. found that only 15.21% of patients in the acute stage had elevated QAlb (20). Previous researches showed that there were several factors that might influence albumin levels in the CSF including age, rate of CSF transport or production, and supraspinal lesions, among others (24, 25). Therefore, QAlb might be an imprecise way to assess BBB dysfunction (16, 17, 24, 26), especially in patients with anti-NMDAR encephalitis. The value of QAlb remains worthy of further study.

In this study, we found CSF IL-6, TNF-α and IL-10 to be increased in anti-NMDAR encephalitis patients in the acute stage. We further investigated the relationship between sCD146 and the proinflammatory cytokines IL-6 and TNF-α, and the anti-inflammatory cytokine IL-10. The results showed that all these three proinflammatory factors were positively correlated with CSF sCD146. In previous studies, abnormal elevation of sCD146 has been reported to correlate with proinflammatory factors, such as TNF-α, IL-13 and IL-17 (5, 27). Early research by our team confirmed that the presence of inflammatory factors in the CSF of anti-NMDAR encephalitis patients reflect the activity of the disease (8, 9, 17, 28–30). In the present study, we considered that local inflammation in the brain was activated, leading to the accumulation of inflammatory factors in the CSF that enhanced the expression of sCD146, ultimately promoting BBB dysfunction. Our results reflected the disturbances in vascular physiology and indicated that the co-activation of these factors might play an important role in the pathogenesis of anti-NMDAR encephalitis. Additional studies are needed to confirm that.

Three months after effective treatment, the levels of sCD146 in CSF of the patients were significantly decreased. Eleven patients (47.8%) recovered well (mRS=1), but seven patients (30.43%) remained with mRS scores above 3 and none had an mRS score of 0, which meant that no patients had achieved a complete recovery in 3 months. Similar research by Titulaer et al. (22) showed that only 53% of patients had symptom improvement within four weeks of first-line immunotherapy. We hypothesized that although inflammation of the brain was partially improved after treatment, BBB dysfunction persisted for a long time because there was long-term high expression of CSF sCD146 and other inflammatory factors. Continued inflammation in the CNS was consistent with the clinical features of slow recovery from anti-NMDAR encephalitis (4).

Here we have shown that the level of CSF sCD146 was positively associated with mRS in the acute stage of anti-NMDAR encephalitis. ROC curve analysis showed that CSF sCD146 ≥33.75 ng/ml can be used as an index to predict the severity of anti-NMDAR encephalitis, and the accuracy of CSF sCD146 in indicating the severity of this disease was remarkable. From this we could infer that the higher the expression of CSF sCD146, the more serious the neurological damage. Our findings implied that CSF sCD146 might hold potential in assessing the severity of anti-NMDAR encephalitis.

Since the initial description of this disease in 2007 (19), the optimal treatment of anti-NMDAR encephalitis has not yet been clarified (21). Previous studies showed that 19.4%–25.5% of anti-NMDAR encephalitis patients remained refractory to current immunotherapies and a significant number of those patients suffered prolonged neurological dysfunctions (21, 22, 31). Since early treatment is closely linked to a better prognosis for patients (22, 32), our study indicates that the short-term immunotherapies we routinely used were effective but may not be comprehensive enough, especially for those patients with persistently high expression of CSF sCD146. It was necessary to strengthen the treatment continuously to relieve the inflammation and facilitate recovery; an early combination with second-line therapy, even novel immunotherapies became particularly important. Therefore, the levels of CSF sCD146 may become a promising indicator for disease monitoring and optimizing clinical treatment decisions in the early stage in anti-NMDAR encephalitis.

Furthermore, it was reported in MS that CSF sCD146 could enhance the expression of adhesion molecules and promote the migration of leukocytes into the CNS (17). Our previous study showed the levels of sICAM-1, sVCAM-1 and sL-selectin in CSF were significantly elevated in anti-NMDAR encephalitis patients. Combined with our findings (30), it may share a similar pathophysiological mechanism in anti-NMDAR encephalitis. We hope to further explore this in our future research.

There were several limitations to our study. First, this was a single-center study, the nature of which may have introduced biases related to a small sample size and a shorter follow-up period. Second, we analyzed CSF and serum samples from anti-NMDAR encephalitis patients but did not conduct further study on pathogenesis.

## Conclusion

In this study, we found that the CSF sCD146 was significant increased in anti-NMDAR encephalitis patients with the acute stage, which were positively correlated with the levels of neuroinflammatory factors and mRS score. These findings suggested that the higher the expression of CSF sCD146, the more severe the neurological damage. Moreover, long-term high expression of CSF sCD146 and other inflammatory factors was accompanied by BBB dysfunction. Therefore, the levels of CSF sCD146 may represent a promising indicator for optimizing clinical treatment decisions in the early stage and disease monitoring in anti-NMDAR encephalitis.

## Data Availability Statement

The raw data supporting the conclusions of this article will be made available by the authors, without undue reservation.

## Ethics Statement

The study was approved by the Ethics Committee of The Affiliated Brain Hospital of Guangzhou Medical University. The patients/participants provided their written informed consent to participate in this study.

## Author Contributions

All authors listed have made a substantial, direct, and intellectual contribution to the work and approved it for publication.

## Funding

This work was supported by Guangzhou Planned Project of Science and Technology (202102080073) and Natural Science Foundation of Guangdong Province (2019A1515011434, 2019A1515011611).

## Conflict of Interest

The authors declare that the research was conducted in the absence of any commercial or financial relationships that could be construed as a potential conflict of interest.
